# Biochemical Characterization of Human Gluconokinase and the Proposed Metabolic Impact of Gluconic Acid as Determined by Constraint Based Metabolic Network Analysis

**DOI:** 10.1371/journal.pone.0098760

**Published:** 2014-06-04

**Authors:** Neha Rohatgi, Tine Kragh Nielsen, Sara Petersen Bjørn, Ivar Axelsson, Giuseppe Paglia, Bjørn Gunnar Voldborg, Bernhard O. Palsson, Óttar Rolfsson

**Affiliations:** 1 Center for Systems Biology, University of Iceland, Reykjavik, Iceland; 2 University of Iceland Biomedical Center, Reykjavik, Iceland; 3 Center for Protein Research, Faculty of Health Sciences, University of Copenhagen, Copenhagen, Denmark; Manchester University, United Kingdom

## Abstract

The metabolism of gluconate is well characterized in prokaryotes where it is known to be degraded following phosphorylation by gluconokinase. Less is known of gluconate metabolism in humans. Human gluconokinase activity was recently identified proposing questions about the metabolic role of gluconate in humans. Here we report the recombinant expression, purification and biochemical characterization of isoform I of human gluconokinase alongside substrate specificity and kinetic assays of the enzyme catalyzed reaction. The enzyme, shown to be a dimer, had ATP dependent phosphorylation activity and strict specificity towards gluconate out of 122 substrates tested. In order to evaluate the metabolic impact of gluconate in humans we modeled gluconate metabolism using steady state metabolic network analysis. The results indicate that significant metabolic flux changes in anabolic pathways linked to the hexose monophosphate shunt (HMS) are induced through a small increase in gluconate concentration. We argue that the enzyme takes part in a context specific carbon flux route into the HMS that, in humans, remains incompletely explored. Apart from the biochemical description of human gluconokinase, the results highlight that little is known of the mechanism of gluconate metabolism in humans despite its widespread use in medicine and consumer products.

## Introduction

Gluconate is a C-1 oxidized derivative of glucose, widely distributed in nature and commonly used as an acidity regulator in both food and drugs [1]. Gluconate is an excellent chelator of calcium ions and calcium gluconate is often given intravenously in order to regulate intravenous Ca^2+^ levels. While this clinical measure undoubtedly focuses on replenishing Ca^2+^, gluconate and its chemical counterpart gluconolactone against which it exists in chemical equilibrium, have in fact been shown to exhibit antioxidant properties and result in increased plasma levels of glutathione [2]. Lowered plasma levels of gluconate have also been associated with Alzheimer's disease [3] and increased oxidative stress [4].

We recently highlighted that gluconate metabolism in humans is unaccounted for using a computational network gap filling approach of the human metabolic network Recon 1. Gluconate catabolism was computed to take place through phosphorylation of gluconate to generate 6-phosphogluconate which could then be further degraded through the hexose monophosphate shunt (HMS) via 6-phosphogluconate dehydrogenase [5]. This catabolic route has indeed been shown to take place in rat liver perfusions [6] and corresponds to well researched degradation routes of gluconate in microorganisms. These involve metabolism via (I) direct internalization from the environment, (II) conversion from L-idonic acid or (III) by direct oxidation of glucose via glucono-1,5-lactone [7]–[9]. A key enzyme in all the gluconate degradation routes is gluconokinase (GntK) which phosphorylates gluconate at the C-6 position thereby priming its catabolism through the HMS or the Entner-Doudoroff pathway in prokaryotes.

**Figure 1:**
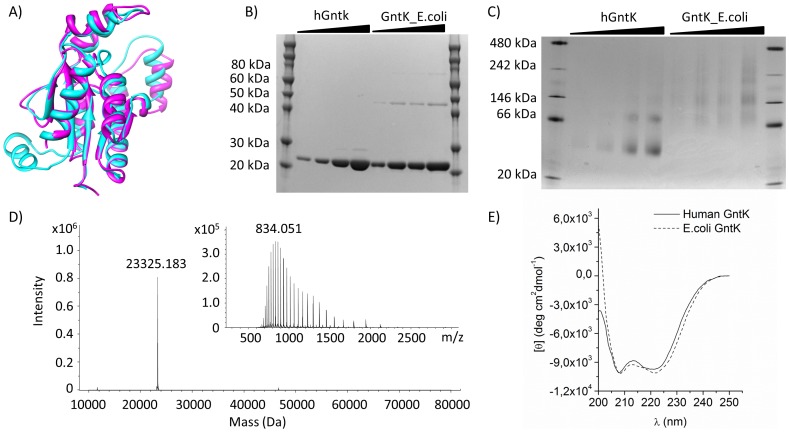
Structural comparison of human GntK to *E.coli* GntK. **A**) An iTasser [44] structural model of human GntK (cyan) superimposed on E.coli GntK (magenta) to which it shows 34% sequence homology. The 18 a.a. insert observed in human GntK is predicted to form a protruding α-helix. **B**) SDS-PAGE of purified human GntK vs. *E.coli* GntK. **C**) Human GntK was observed to oligomerize as multiples of protein dimers. **D**) The ionization pattern (inset) and deconvoluted mass spectrum of purified human GntK **E**) Circular dichroism spectra of the two proteins are indicative of a similar tertiary structure.

The human gene C9orf103 was identified through a metabolic network gap filling effort of Recon 1 and through amino acid sequence alignment as a likely kinase responsible for the initial step in gluconate catabolism in humans [5]. C9orf103 had previously been cloned and sequenced in relation to it being a plausible tumor suppressor gene associated with acute myeloid leukemia [10]. *In vitro* assays of isoforms I and II of C9orf103 expressed in human HeLa cell lysates showed that only isoform I had ATP dependant phosphorylation activity consistent with the absence of a phosphate binding loop domain in isoform II. Isoform I shows 35% sequence similarity to both GntKs encoded within the *E.coli* genome. A defining structural difference is an 18 amino acid insert that is found in various NMP kinases that have similar protein structure to *E.coli* GntK and of which many are known and act on a broad variety of substrates [5], [11] (**Figure 1A**).

The presence of a functional human GntK is interesting. Publically available expression and proteomic profiling datasets show that human GntK is differentially expressed in the thyroid and brain amongst others tissues [12], [13]. This implies an unknown or expanded role for the protein outside its proposed catabolic role in the mammalian kidney and liver. Direct oxidation of glucose to generate gluconate is generally not perceived to take place in humans where phosphorylation precedes the oxidization step. Excluding dietary origins, the metabolic origins of gluconate remain unknown in humans. The notion that GntK activity is encoded within the human genome however highlights a carbon flux route into the HMS with possible implications for human energy metabolism given the central role that the pathway plays in fatty acid, nucleotide and amino acid synthesis and in combating oxidative stress [14]. In order to further characterize human GntK and gluconate metabolism, we report the expression, kinetic characterisation and substrate specificity of a recombinant human GntK isoform I expressed in *E.coli*. Furthermore, we assessed the contribution of gluconate to human central metabolism through constraint based modeling of the curated erythrocyte metabolic model iARB-RBC-283 [15] and discuss its effect on human metabolism.

## Experimental

### Expression and purification

Cloning expression and purification of C9orf103 was carried out at the Center for protein research at the University of Copenhagen. C9orf103 isoform I (BI826543, IMAGE ID: 5168613) was inserted into an in-house expression vector pCPR0011 downstream of a 6xHIS and STREPII affinity tags generating pCPR_GNTK-1 which, following sequence confirmation, was transformed into Rosetta cells. The clone was inoculated in 50 ml of TB media with kanamycin (50 µg/ml) and chloramphenicol (25 µg/ml) and incubated overnight at 37°C at 180 rpm. 7 ml of overnight culture was added to 750 ml of media with kanamycin (50 µg/ml), and expression induced with 0.5 mM IPTG at approximately OD_600_ = 1.5. Cells were then incubated further at 18°C overnight and harvested by centrifugation at 4000 g for 10 min (culture OD_600_ = 13.5 at harvest). A 4.5 L culture resulted in a 110 g cell pellet that was stored at −20°C until purification at which point it was thawed, resuspended in 400 ml of running buffer (50 mM sodium phosphate, 0.3 mM NaCl, 10 mM imidazole, 10% glycerol and 0.5 mM TCEP at pH 7.5) containing protease inhibitors and benzonase, and lysed using french press. The lysate was cleared by centrifugation at 18000 g for 30 min and filtered through a 0.22 µm filter and run through a Nickel HiTrap column at a flow rate of 0.8 ml/min. The protein was eluted off the column with running buffer containing 0.5 M imidazole. Following dialysis and concentration, the eluate was further purified using a 120 ml HiLoad 16/60 superdex 200 size exclusion column eluted with 50 mM sodium phosphate, 150 mM NaCl, 10% glycerol, 0.5 mM TCEP, pH 7.5. 2 ml fractions were collected and samples pooled affording 14 ml of 5.8 mg/ml protein using a calculated molar absorption coefficient of 22461 Lmol^−1^cm The enzyme was aliquoted and stored in −80°C in 50 mM sodium phosphate, 150 mM NaCl, 10% glycerol and 0.5 mM TCEP at pH 7.5. PAGE was carried out on NuPAGE 12% Bis-tris gels and NativePAGE 4–16% Bis-Tris Gels using Novex Sharp pre-stained protein markers and NativeMark unstained native markers as size markers, respectively. Protein aliquots were removed from −80°C and thawed on ice prior to enzyme assays. Loss of activity was not observed after storage at 4°C for two weeks.

### Circular Dichroism

Samples containing human GntK enzymes were dialyzed into 100 mM phosphate, 40 mM NaCl, 2.5 mM MgCl_2_ pH 7.2 and a A J-810 CD spectrometer from Jasco (Tokyo, Japan) was used to obtain CD spectra from 190 to 260 nm. For the Tm experiments, the CD signal at 222 nm was monitored as the temperature increased by 1°C/min. The protein concentration was in the range 0.05–0.1 mg/mL.

### Kinetic assays

The rate of gluconate phosphorylation was evaluated by coupling GntK activity with that of 6-phosphogluconate oxidation by 6-phosphogluconate dehydrogenase (6PGDH, Megazyme) and monitoring the absorbance increase at 340 nm over time corresponding to the accumulation of NADPH. Assays were carried out in 100 mM phosphate, 40 mM NaCl, 2.5 mM MgCl_2_ pH 7.2 (Buffer A) taking into account assays reported by Leder [16]. Glukonokinase was kept at 0.25 µM. 0.03 units of 6PGDH were used per reaction. ATP final concentration was 1 mM and gluconate concentration varied from 0.1–10 mM in order to determine gluconate kinetic parameters but vice versa for ATP. NADP^+^ concentration was 0.65 mM. Reactions were initiated by addition of the target substrate. Reactions were carried out in 96 well clear, flat bottom plates and absorbance values were correlated to NADPH concentration by extrapolation off a freshly diluted NADPH standard curve. Initial reaction rates were obtained from reaction progress curves and plotted against substrate concentration and the resulting data fit to the Michaelis Menten model using Softmax Pro. The values shown represent average values from six biological replicates, performed in technical triplicate.

### Substrate specificity assays

Reactions were carried out in 96 well plates in buffer A with ligand concentration at 0.5 mM, ATP at 0.1 mM and human GntK at 250 nM in 50 µl total volume. Following a 30 min incubation time at room temperature, ATP dependant phosphorylation of substrates by human GntK was estimated by measuring the drop in ATP concentration using the Promega Cell Titer Glo assay according to the manufacturers' protocol. Data is portrayed as the difference in luminescence obtained for a substrate from the average luminescence of all compounds. **File S1** gives luminescence values in each reaction subtracted from no GlcnK control luminescence along with calculated z-scores and p-values for each compound [17].

### Modelling the uptake of gluconate metabolism in erythrocytes

Modelling of gluconate metabolism was done using the erythrocyte metabolic model iARB-RBC-283 [15] available through the biomodels database as MODEL1106080000. iARB-RBC-283 is a genome scale metabolic model generated and curated by mining red blood cell literature and red cell proteomic datasets. It represents the most complete representation of human red cell metabolism in SBML format and is comprised of 283 genes, 267 unique small metabolites and 469 reactions. Gluconate was introduced into the iARB-RBC-283 model by adding three reactions, namely, for exchange, transport and for conversion of D-gluconate to 6-phospho-D-gluconate using the COBRA toolbox [18]. It is important to state that addition of the exchange and transport reactions are only done in order to introduce gluconate into the metabolic network so that the effect of varying intracellular gluconate concentration on metabolism can be modeled. Flux variability analysis (FVA) was utilized to determine the changes in flux in reactions within the network. FVA calculates the maximum and minimum possible flux through each reaction under the given constraints [19]. A reaction was considered to be effected if the change in the flux range upon gluconate consumption was equal to or more than 40% of the flux range in the absence of gluconate [15]. The flux range is the absolute difference between the maximum and minimum fluxes of a reaction. Flux variability analysis results are given in **File S1** along with calculated shadow prices of each reaction in iARB-RBC-283 in the presence and absence of the added gluconate reactions. Shadow prices are indicator of how a metabolite affects the set objective, which is to be maximized or minimized [20]. Flux balance analysis, following relaxation of each transport reaction bound are also given. All analysis of metabolic network properties were carried out using the COBRA toolbox v.2.0 [18]. Abbreviations of all metabolites are according to the BiGG database [21].

## Results

### Characterisation of human GntK

Human GntK was over expressed and purified as described in materials and methods. The vector construct expressed a protein product of 208 a.a. compared to the 187 a.a. residue wild type protein due to N-terminal hexahistidine and strep tags. SDS-PAGE showed the protein to be more than 95% pure and migrated to a similar extent as commercially available *E.coli* GntK (**Figure 1** **B**). Both of these were immunogenic towards a polyclonal antibody against human GntK. The major band migrated with an observed molecular mass of 21,825±0.7 Da having a calculated mass of 23,326 Da. This corresponds well with the mass spectrometry result of 23,325.2 Da (**Figure 1C**). Native gel electrophoresis showed that the protein exists as dimers and oligomerizes as such. The calculated molecular weight of the two most prominent bands in lanes 4 and 5 in **Figure 1D** were 41.785±1.9 kDa and 85.03±3.3 kDa corresponding to a GntK dimer and tetramer. The *E.coli* GntK was similarly observed to oligomerize as multiples of dimers and has been shown to exist as a homodimer in solution [8]. The circular dichroism spectra, an indicator of protein secondary structure [22], of the recombinant forms of both GntKs was similar. Defining negative bands at 208 nm and 222 nm were observed indicative of an α-helical structure although differences were seen in their intensities hinting at conformational differences. A negative band was observed in the human GntK at 200 nm which was not observed in the *E.coli* GntK a difference that was accredited to the unstructured N-terminal region containing the His and Strep tags.

### Kinetics of GntK

**Figure 2:**
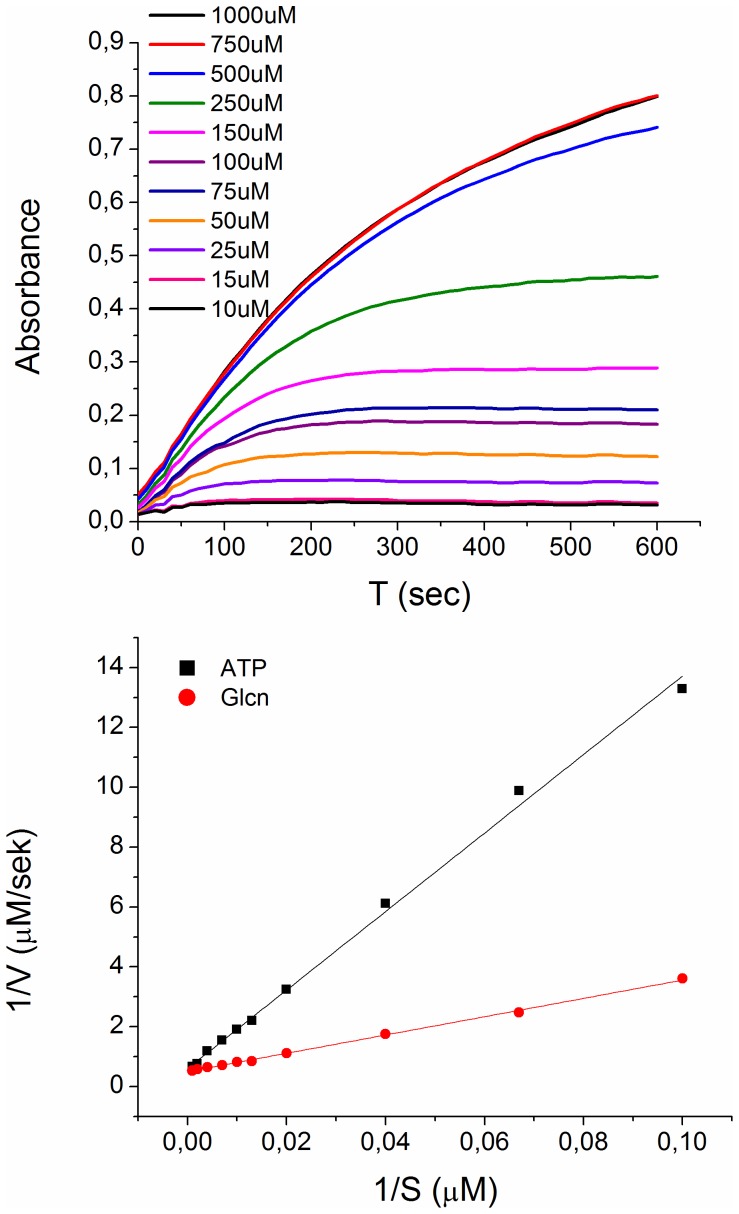
Reaction kinetics of the phosphorylation of gluconate by human GntK. **A**) Reaction progression curves at increasing gluconate concentration. Initial rates were plotted against substrate concentrations and fit to the Michaelis Menten model using nonlinear regression to afford kinetic parameters shown in **Table 1**. **B**) Double reciprocal representation of the kinetic data.

The steady state kinetics of GntK was investigated by coupling 6-phosphogluconate formation to consumption by 6-phosphogluconate dehydrogenase and measuring the resulting increase in NADPH formation spectrophotometrically at 340 nm (**Figure 2**). Initial rates were calculated and plotted against substrate concentration and the resulting data fit to the Michaelis Menten equation. A double reciprocal plot of initial reaction rates at varied gluconate and ATP concentrations, respectively, is shown in **Figure 2B**. The experimental kinetic parameters for phosphorylation of gluconate are given in **Table 1**. The affinity of the GntK for gluconate was roughly 3 times that for ATP while the turnover numbers for both substrates were the same. Phosphorylation of gluconate by the nucleoside triphosphates GTP, CTP and UTP was observed. The kinetic parameters for these were not accurately determined. Initial rate data however implied that the Michaelis constant for UTP is roughly five orders of magnitude higher than that of ATP and ten orders higher for GTP and CTP. Nucleotide hydrolysis in the absence of gluconate was not detected.

**Table 1:** Human GntK kinetic properties.

	*K*_m_ (µM)	*V*_max_ (µM/s)	*k*_cat_ (s^−1^)	*k*_cat_/*K*_m_ (s^−1^µM^−1^)	R^2^
Gluconate	106.4±18.5	2.3±0.1	9.3±0.5	0.089±0.013	0.98±0.008
ATP	343.8±13.9	2.4±0.1	9.5±0.5	0.028±0.002	0.99±0.002

### Substrate specificity of GntK

**Figure 3:**
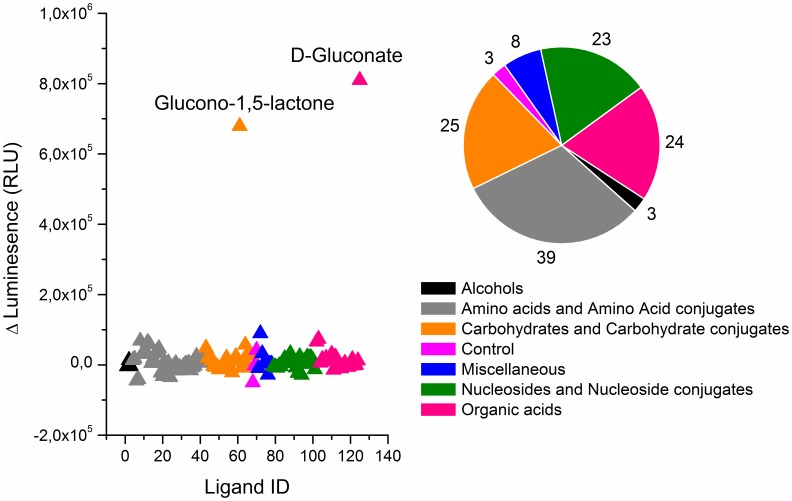
Human GntK showed strict substrate specificity for D-gluconate out of 122 common metabolites involved in human core metabolism. Activity was only observed for D-gluconate and its lactone derivative. These had calculated p-values of 9.5×10^−17^ and 3.4×10^−12^ based on Z-scores of −8.2 and −6.7 respectively. The pie chart shows the number of compounds tested according to their taxonomy class.

The annotation of C9orf103 encoding GntK activity was tested further by investigating substrate dependent ATP phosphorylation against an in-house metabolic library composed of a broad spectrum of metabolites involved in core metabolism (**Figure 3**). Following incubation of human GntK with the substrates at a fixed ATP concentration, the drop in ATP concentration was detected spectrophotometrically. The enzyme showed little activity above background for any of the substrates tested apart from gluconate and glucono-1,5-lactone. Included in this list were twenty five carbohydrate/carbohydrate conjugates and twenty four organic acids, including known substrates of closely related carbohydrate kinases [23] such as D-ribulose, meso-erythrose and glycerol. A full list of all compounds screened is given in **File S1**.

### The effects of gluconate on core metabolism

**Figure 4:**
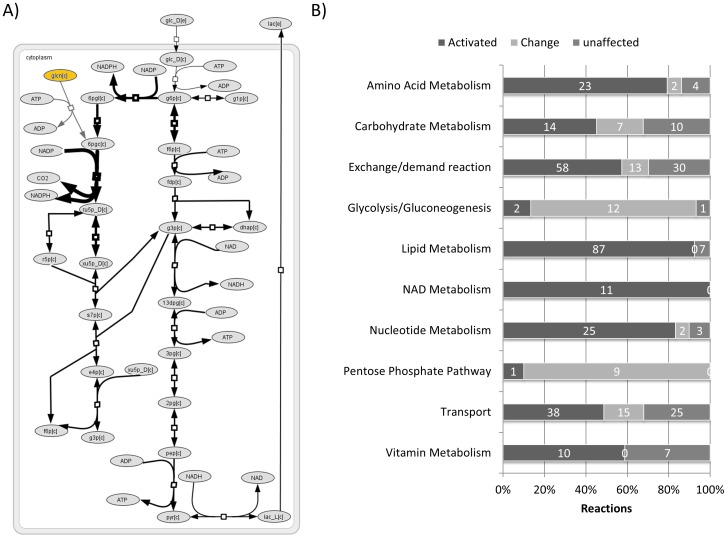
Modelling the contribution of gluconate to core metabolism. **A**) Metabolic flux through glycolysis and the HMS is affected upon gluconate (yellow) consumption via GntK. The two added reactions (grey) feed gluconate into the HMS as described in the text. Reactions in the HMS and glycolysis with changed flux ranges upon gluconate uptake are indicated by arrow thickness as defined in **File S1**. Metabolite abbreviations are consistent with the BiGG database. **B**) Flux variability analysis showed changes in the flux ranges of 329 out of the 416 reactions in the RBC model upon incorporation of gluconate. 56 miscellaneous reactions, which were not defined properly in the model, have been removed from this graph to simplify the graph. Gluconate degradation also activated reactions that otherwise did not carry flux due to constraints imposed by flux into the HMS through glucose-6-phosphate dehydrogenase which was floated at physiological 5–10% of glucose uptake.

Flux variability analysis of the erythrocyte metabolic model iAB-RBC-283 [15] was utilized in order to evaluate changes in cellular core metabolism induced by gluconate degradation. Gluconate was fed into the HMS via 6-phosphogluconate by relaxing the bounds on exchange for gluconate at a fixed glucose uptake rate of 1.12 mmol/gDW/h [24]. This resulted in a 0.06 mmol/gDW/h flux increase through the ATP dependent Na^2+^/K^+^ pump that was set as the objective function. The flux through GntK spanned 0.20–4.46 mmol/gDW/h. An increase in the flux range of all reactions in both the HMS and glycolysis was also observed upon uptake of gluconate (**Figure 4a**). Accordingly, production of NADPH increased that in turn increased production of reduced glutathione and flux through reactions associated with fatty acid metabolism in the model. As glycolysis is interconnected to the HMS, this also led to increased pyruvate and lactate production. In fact, flux ranges for roughly 80% of all the reactions in the metabolic network were perturbed upon gluconate metabolism by more than 40% of the initial flux range in the absence of gluconate. Out of the reactions showing a change in flux ranges, multiple reactions were activated in the presence of gluconate meaning that no flux through these reactions takes place prior to gluconate metabolism in the metabolic model (**Figure 4b**).

**Figure 5:**
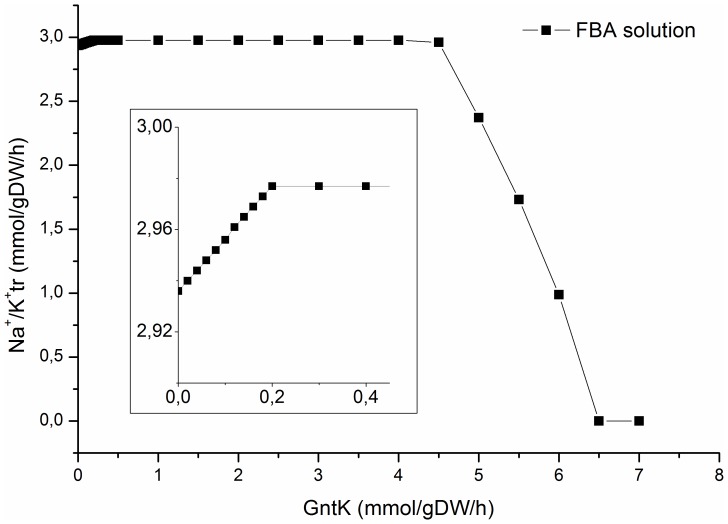
Robustness analysis of gluconate flux through GntK vs. the Na^2+^/K^+^ transporter pump set as the objective in iAB-RBC-283. A slight increase in flux through the Na^2+^/K^+^ transporter pump that then became constant up to a gluconate flux rate of 4.5 mmol/gDW/h was observed upon gluconate addition. This resulted in an increase (defined as >40%) in the flux range of multiple reactions in iAB-RBC-283. At GntK flux rates above 4.5 mmol/gDW/h bicarbonate exchange becomes limiting and as a result flux through the Na^2+^/K^+^ transporter pump drops. The figure inset is the same as the above but with a modified scale.

The effect of GntK flux on the objective was investigated further employing robustness analysis. Flux balance analysis was performed at forced and increasing rates (0–20 mmol/gDW/h) of gluconate uptake, at a fixed glucose uptake rate of 1.12 mmol/gDW/h [24] (**Figure 5**). As expected, based on the FVA analysis, the objective maximum was reached at a flux of 0.2 mmol/gDW/h through GntK, which is roughly 18% of the set glucose uptake rate. Increasing the GntK flux rate past 4.5 mmol/gDW/h resulted in a sharp drop in the objective as metabolites required for gluconate catabolism became limiting due to physiologically determined model constraints [24]. In order to pinpoint these metabolites we calculated shadow prices for each metabolite in the metabolic network in the presence and absence of gluconate (**File S1**). In the absence of gluconate, 89 out of the 267 unique metabolites within iAB-RBC-283 had an effect on flux through the ATP dependent Na^2+^/K^+^ pump while in the presence of gluconate this number was reduced to 10. These metabolites were all ultimately associated with either bicarbonate transport/exchange (bicarbonate, chloride, potassium, bilirubin monoglucuronide, bilirubin, biliverdin and carbon monoxide) or glutathione synthesis (L-cysteine, γ-L-glutamyl-L-cysteine and glycine) and became limiting on the account of increased levels of NADPH and carbon dioxide generated from gluconate catabolism.

Further investigation into the limiting factor of gluconate metabolism was performed by relaxing bounds constraining each exchange reaction followed by flux variability analysis (**File S1**). In the absence of gluconate, a total of 7 reactions, including exchange of glucose, galactose, glucosamine mannose, fructose, inosine and adenosine were observed to affect the objective function. In the presence of gluconate however there was only one reaction, namely bicarbonate exchange. The modeling results indicate that metabolism of gluconate via the HMS compensates for the aforementioned metabolites. This occurs until transport of bicarbonate, generated from increased levels of carbon dioxide that in turn originate from increased dehydrogenase activity, limit metabolism.

## Discussion

Gluconate metabolism is well characterized in microorganisms due to bioengineering efforts to enhance production of gluconate for industry. In humans, studies have been carried out that have shown the compound to be safe and degraded via core metabolic pathways [25]. Crucially, Stetten demonstrated that gluconate is metabolized differently than glucose in rats and contributes to glycogen and CO_2_ production [6]. In humans however gluconate metabolism remains under-explored in light of its potential role in metabolism. The molecular details of gluconate metabolism and how it might contribute to energy metabolism have not been characterized in humans. Here we employed a combined approach composed of traditional molecular biology and computational network modeling methods to gain information on GntK and gluconate metabolism in humans, respectively. We characterized isoform I of human GntK in terms of structure, kinetic properties and substrate specificity. Metabolic network analysis, designed to afford ideas on the contribution of gluconate to core metabolism was performed on the genome scale metabolic network iAB-RBC-283 encompassing red cell metabolism. These show that combined glucose and gluconate metabolism is likely to result in beneficial effects on cellular anabolism.

Isoform I of human GntK was characterized in terms of structure, kinetic properties and substrate specificity. Denaturing gel electrophoresis and mass spectrometry confirmed the purity and identity of the recombinant enzyme which was shown to conform to a dimer structure using native gel electrophoresis (**Figure 1**). Indeed, many of the carbohydrate kinases including, glycerol kinase, shikimate kinase, xylulokinase and GntK are known to be active as dimers [23] and this appears to be the case for human GntK as well. The 18 a.a. insertion found in human GntK which is likely the defining structural difference between human GntK and prokaryotic GntK, does therefore not affect the dimerization properties of the enzyme. This corresponds with the electronic structural proposal of human GntK indicating that the insert is located away from the dimer interaction interface of the protein. We obtained CD spectra, a method for determining the secondary structure of proteins [22]. The CD spectra of GntK and *E.coli* GntK provided further confirmation of structural similarities and suggested that the enzyme most likely adheres to the α/β structure and topology solved for *E.coli* GntK. More focused structural biology methods are however required to deduce the fine details of the structure of human GntK.

Apart from non-cognate GntKs, human GntK shows sequence similarity to various NMP kinases known to catalyze the phosphorylation of a broad range of substrates [11]. We found that recombinant human GntK showed strict substrate specificity for gluconate. ATP dependant phosphorylation activity was also observed for glucono-1,5-lactone as it exists in equilibrium with gluconate in aqueous conditions. The enzyme was inactive against the hexoses D-glucose, D-galactose, D-fructose, D-mannose and D-glucosamine. Similarly no activity was observed against the pentoses D-ribose, D-ribulose, D-arabinose, D-xylulose, D-xylose and D-erythrose. Finally no activity was observed against various polyols and carboxylic acids which differ from gluconate in terms of carbon chain length and in their degree of saturation. Previously, for hog GntK, substrate specificity analyses were focused on compounds similar in structure to gluconate. The substrates tested in this study were structurally more diverse. These results refute automated electronic annotations of this enzyme having shikimate kinase and adenylate kinase activity and imply that the phosphorylation activity of human GntK is indeed specific for gluconate. This is in line with the findings for hog GntK [16] and the constraints on substrate binding implied from the crystal structure solved for *E.coli* GntK [11].

The estimated Michaelis constant obtained for human GntK towards gluconate was similar to that found for hog GntK, *K*_m_ = 140 µM, but higher than those reported for *E.coli* GntK, *K*_m_ = 20 µM [8], [9]. The ratio of *K*_mglcn_/*K*_mATP_ is however similar between human GntK and *E.coli* GntK, 3.2 vs. 3.0 and implies that the reaction dynamics are similar for these enzymes. The turnover numbers for both gluconate and ATP were nearly identical (**Table 1**) however the catalytic efficiency (*k*_cat_/*K*_m_) for gluconate was slightly higher than for ATP indicative of a sequential reaction mechanism. The order of substrate binding was however not determined. The reaction mechanism for GntK from *E.coli* and *S. pombe* have been proposed, and while both found that the reaction takes place via a transition state following sequential binding, the binding order of substrates was proposed to be ordered for *E.coli* GntK as opposed to random for *S.pombe* GntK. The results reported here do not allow the reaction mechanism for human GntK to be determined. A detailed report on the kinetics and thermodynamics of the human GntK reaction will be reported separately.

The only reported degradation route of gluconate in vertebrates is via the HMS [16], [26], [27]. In order to assess how gluconate contributes to global metabolism, its degradation through the HMS was modeled using FVA of the erythrocyte metabolic model iAB-RBC-283. Although human genome scale cell and tissue specific metabolic models have been generated for a variety of context specific applications [28]–[32], this model was chosen to model metabolism for several reasons. Firstly the model is small with few components, uncluttered with minimum possibilities of false positives introduced by metabolic gaps, dead ends and flux loops [30], [33], [34] and the model has defined metabolite exchange constraints [15]. Secondly the Na^+^/K^+^ pump is a realistic objective function, the maintenance of the Na^+^ concentration gradient has been shown to account for up to 70% of cell ATP expenditure [35]. Finally, erythrocytes have a very active HMS so any observed flux changes were likely to be emphasized with a minimum of false positives. A drawback of this model is the absence of core metabolic pathways downstream of glycolysis namely the TCA cycle and oxidative phosphorylation that are indeed central to cells that carry out aerobic glycolysis.

Introducing gluconate had an effect on flux through reactions within the model. 269 reactions were activated and 60 reactions showed changes in their flux range out of a total of 472 reactions in iAB-RBC-283. In the HMS, 9 out of 10 reactions had increased flux ranges, however the biggest effect was observed in metabolic pathways involving metabolites/reactions derived from the HMS. These included nucleotide, carbohydrate, amino acid and lipid metabolic pathways due to increased levels of ribose-5-phosphate, xylulose-5-phosphate, erythrose-4-phosphate and NADPH, respectively. This suggests that the metabolic repertoire/capacity of the model is significantly increased upon gluconate degradation. Increasing flux through the HMS in the form of gluconate relieves constraints on these pathways imposed by the glucose uptake rate and the HMS split ratio. Indeed a similar effect would be observed if the flux into the HMS would be increased through glucose-6-phosphate dehydrogenase. In erythrocytes glucose-6-phosphate deyhydrogenase is the main source of NADPH and is the most common enzyme deficiency known and has deleterious effects when homozygous [36]. The modeling results here simply demonstrate that considerable control of key metabolic pathways can be achieved by bypassing this regulatory enzyme into the HMS. There is little apparent energetic gain to feeding the HMS through GntK or through hexokinase. Both pathways supposedly result in the same use and production of ATP and NADPH. We propose that GntK or enzymes upstream of it are involved in context specific regulation of carbon flux into the HMS. A gene co-expression analysis or knockdown study of the effect of GntK on core metabolism could help shed light on a novel flux route into the HMS in humans.

There are several ways in which gluconate could be generated endogenously. It could be formed by spontaneous oxidation of glucose, as a product of advance glycation end-product formation [37], as a product of microbiota [38], through undiscovered anabolism of complex glycans or simply by dephosphorylation of 6-phosphogluconate. In these cases GntK might function as a salvaging enzyme, re-routing gluconate back into central carbon metabolism through the HMS. Another route of gluconate formation is its production through direct oxidation of glucose via glucose dehydrogenase (EC 1.1.1.47) and gluconolactonase (EC 3.1.1.17). Glucose dehydrogenase activity is not generally believed to be a central part of human metabolism. Although the endoplasmic reticulum localized hexose-6-phosphate dehydrogenase has been reported to carry out the direct oxidation of glucose, generating D-glucono-1,5-lactone, its main substrate is glucose-6-phosphate [39], [40]. D-gluconolactonase activity, affording gluconate from D-glucono-1,5-lactone, has been shown to be encoded by the microsomal enzyme SMP30 in the context of ascorbic acid synthesis in rats [41]. Given that humans do not synthesize ascorbic acid it is likely that SMP30 has a different metabolic role in humans. In an attempt to address this, over expression of SMP30 in HEPG2 cells has been shown to result in significant reduction in reactive oxygen species with associated decreases in lipid peroxidation levels and superoxide dismutase activity. The effects were attributed to intracellular [Ca^2+^] modulation brought about by increased SMP30. SMP30 itself was shown not to have radical scavenging ability and *Handa et al.* proposed an indirect antioxidant ability of SMP30 [42]. In light of our modeling results, which indicate how human GntK activity affects the global metabolic phenotype, we propose that the effect observed upon SMP30 over expression and later complemented by knockdown experiments of SMP30, is brought about by modulating flux into the HMS through gluconate thereby promoting NADPH formation.

Herein we have described the biochemical characterization of human GntK, an enzyme flagged in two network gap filling analyses of Recon 1 [5], [43] and employed constraint based network analysis to assess how gluconate might impact human metabolism. The results advance knowledge of the biochemical properties of isoform I of human GntK and suggest that considerable perturbation of metabolic pathways associated with the HMS result given that gluconate degradation follows similar routes in humans as reported in vertebrates. Furthermore, these data serve to highlight an overlooked carbon flux pathway into the HMS in humans which could be of significance given the pathways central anabolic role and importance in combating oxidative stress. Finally the results demonstrate the application of human metabolic models to assess metabolic phenotypes and how these can be put into context with existing biological data to advance functional genomic hypotheses.

## Supporting Information

File S1 The file contains five worksheets that report the following 1: measured luminesence values from the substrate specificity assays 2: results from flux variability analysis of in iAB-RBC-283 with and without gluconate. 3: how flux values relate to arrow thickness as portrayed in figure 4. 4: Calculated shadow prices in the presence and absence of gluconate. 5: FBA solutions obtained upon relaxing transport/exchange flux boundaries in the presence and absence of gluconate.
(XLSX)
